# Unraveling the relationship between anoikis-related genes and cancer-associated fibroblasts in liver hepatocellular carcinoma

**DOI:** 10.1016/j.heliyon.2024.e35306

**Published:** 2024-07-29

**Authors:** Meng Sun, Jiangtao Bai, Haisong Wang, Mei Li, Long Zhou, Shanfeng Li

**Affiliations:** Department of Interventional Vascular Surgery, Affiliated Hospital of Hebei University, Baoding, China

**Keywords:** Anoikis, Cancer-associated fibroblasts, Immune cell infiltration, Prediction model

## Abstract

This study intended to determine the molecular subtypes of liver hepatocellular carcinoma (LIHC) on the strength of anoikis-related genes (ARGs) and to assess their prognostic value and prospective relationship with immune cell infiltration and cancer-associated fibroblasts (CAFs). Univariate Cox regression analysis yielded 66 prognosis-related ARGs and classified LIHC into two distinct subtypes, with subtype A demonstrating overexpression of most prognosis-related ARGs and a significant survival disadvantage. Furthermore, a reliable prediction model was developed using ARGs to evaluate the risk of LIHC patients. This model served as an independent prognostic indicator and a quantitative tool for clinical prognostic prediction. Additionally, subtype-specific differences in immune cell infiltration were observed, and the risk score was potentially linked to immune-related characteristics. Moreover, the study identified a significant association between CAF score and LIHC prognosis, with a low CAF score indicating a favorable patient prognosis. In conclusion, this study reveals the molecular mechanisms underlying the development and progression of LIHC and identifies potential therapeutic targets for the disease.

## 1 Introduction

Hepatocellular carcinoma (HCC) represents the most frequently occurred primary malignancy of the liver and causes significant cancer-associated mortality worldwide. Owing to the absence of specific symptoms, the majority of HCC patients suffer from a late diagnosis and poor prognosis. Against this backdrop, the identification of specific prognostic biomarkers for HCC contributes to developing personalized therapies for HCC patients and improving their prognoses [1].

Anoikis is a programmed cell death mechanism that occurs as a loss of correct cell-extracellular matrix (ECM) arises, a structural network promoting cell function and tissue integrity [[2], [3], [4]]. Anoikis resistance, a defining hallmark of cancer, enables cancer cells to survive and flourish outside their native tissue environments, leading to metastasis and drug resistance [2,5]. Some anoikis-related genes (ARGs) dysregulated in HCC have been revealed [6,7], indicating the potential of utilizing these ARGs as prognostic biomarkers to predict HCC patient survival.

Cancer-associated fibroblasts (CAFs), as the main stromal component in the tumor microenvironment, perform a crucial role in the growth and metastatic processes of tumors. These cells actively engage in cancer progression *via* extensive reciprocal signaling interactions with cancer cells in the microenvironment [8,9]. A high content of CAFs has been linked to a poor prognosis for multiple cancers, including HCC [10]. A recently developed CAF scoring system has shown favorable performance in predicting the outcomes of HCC patients, demonstrating its potential as a prognostic biomarker.

The complex interaction between the immune system and cancer has been well-established [11]. Immune cells within the tumor microenvironment exert both pro-tumorigenic and anti-tumorigenic impacts on HCC [12,13]. Therapies targeting the immune system have emerged as a promising treatment modality for HCC, which requires an accurate prediction of the immune reaction in HCC patients.

Herein, we intended to investigate the promising predictive value of ARGs and CAF scores for immune response in HCC. We hypothesized that dysregulated ARGs and elevated CAF scores were correlated with decreased immune responses in HCC patients, leading to compromised prognoses. Our findings may have significant clinical implications. It is expected to develop personalized treatment strategies based on the immune response and tumor microenvironment of HCC patients. The overall survival of HCC sufferers and their quality of life are promising to be improved by optimizing prognosis prediction and treatment selection.

## 2 Methods

### 2.1 Data collection

The liver hepatocellular carcinoma (LIHC) gene expression data and clinical data (GSE76427) [14] were searched. All TCGA-LIHC data, containing mutation, copy number variation (CNV), mRNA expression, and clinical data, were acquired from The Cancer Genome Atlas (TCGA) database. A total of 640 ARGs were obtained from GeneCards (https://www.genecards.org/) and Harmonizome (https://maayanlab.cloud/Harmonizome/), of these genes were screened with a score >0.4 (Table s1). Then, the R package "limma" was utilized for differential analysis of the aforementioned ARGs with standard thresholds (p-value ≦ 0.05, logFC >1). Since all data are publicly available, additional ethical sanction was waived for this study.

### 2.2 Identification of two subtypes of LIHC with ARG expression

Unsupervised consensus clustering was applied to cluster LIHC patients via the "ConsensusClusterPlus" R package [15]. Following that, principal component analysis (PCA) was implemented with the application of the R package "ggplot2" [16]. Through the "survival" and "survminer" R packages, Kaplan-Meier analysis was conducted for analyzing survival differences between clusters. Additionally, single-sample gene set enrichment analysis (ssGSEA) was adopted to evaluate the immune infiltration of LIHC for 24 types of immune cells in the two clusters. Furthermore, functional analyses, including GSEA [17] and gene set variation analysis (GSVA) [18], were carried out with the assistance of "GSEA" and "GSVA" R packages, respectively.

### 2.3 Construction of anoikis-related prognostic risk model

The prognostic ARGs were determined using uni-variable Cox regression and LASSO Cox regression analyses utilizing the "glmnet" R package [19]. As a consequence of optimizing the model, the risk score equals the sum of each gene's expression divided by the coefficient: $Risk\;score=\sum_{i=1}^{n}\text{Coefi}\times \text{Xi}$. Where, Coefi signifies the correlation coefficient and Xi denotes the expression of the prognostic genes. We used the "timeROC" package in R software for receiver operating characteristic (ROC) curve analyses. We employed the "rms" R package to develop a predictive nomogram with clinical characteristics and risk scores. Also, the "CIBERSORT" R package was employed to determine the proportions of immune cell types in either the low-risk or high-risk population [20]. We also conducted Pearson correlation analysis to examine the correlation between risk and immune-related scores. Conclusively, the drug-imputed sensitivity score from Sanger's Genomics of Drug Sensitivity in Cancer (GDSC) was computed using the "oncoPredict" package [21] to determine the therapeutic impact of drugs on LIHC patients.

### 2.4 CAF score analysis

We then estimated CAF scores by four algorithms (EPIC, xCell, MCPcounter, and estimate) using TCGA-LIHC data [22]. The modules of highly relevant genes based on CAF scores were identified through co-expression analysis with the utilization of the weighted gene correlation network analysis (WGCNA) R package. Furthermore, we classified patients into high-risk and low-risk sets through tumor immune dysfunction and exclusion (TIDE) analysis to evaluate the response of LIHC patients to immunotherapy. Ultimately, the association between tumor mutational burden (TMB) and CAF scores was discussed using the R packages "maftools" [23] and "reshape2".

### 2.5 Immune cell-cell interactions in the LIHC tumor microenvironment

Single-cell dataset (GSE146115) was collected from the GEO website [24]. After quality control (QC) by the R package "Seurat" [25], cell populations were classified based on marker genes. The main immune cell types were determined based on their respective markers. The t-distributed stochastic neighbor embedding (t-SNE) was employed for dimension reduction to represent the cell types visually. According to gene expression profiles included in single-cell RNA sequencing (scRNA-seq) data, the intercellular communication networks can be quantitatively analyzed and inferred with the "CellChat" R package [26]. CellChat features a comprehensive database of receptor-ligand interactions. For the most critical intercellular interactions in the LIHC tumor microenvironment, receptor-ligand pairs relevant to hub genes were examined for further investigation, which enabled us to identify the possible immune cell-cell interactions, promoting the understanding of their communications with the microenvironment.

### 2.6 Statistical analysis

The R packages adopted here consisted of “limma”, “pheatmap”, “ConsensusClusterPlus”, “ggplot2”, “enrichplot”, “clusterProfiler”, “ggpubr”, “survminer”, “timeROC”, “glmnet”, "GSVA","rms", "CIBERSORT","oncoPredict", "maftools", "reshape2", "Seurat", and "CellChat". To determine the significance of statistical data derived from our research, one-way analysis of variance (ANOVA), unpaired two-tailed *t*-test, and Kaplan-Meier analysis were carried out. The statistical processing of all data was realized using R (version 4.1.0) software. The significance level (P-value) P < 0.05 was considered statistically significant.

## 3 Results

### 3.1 ARGs-based identification of molecular subtypes of LIHC

**Fig. 1:**
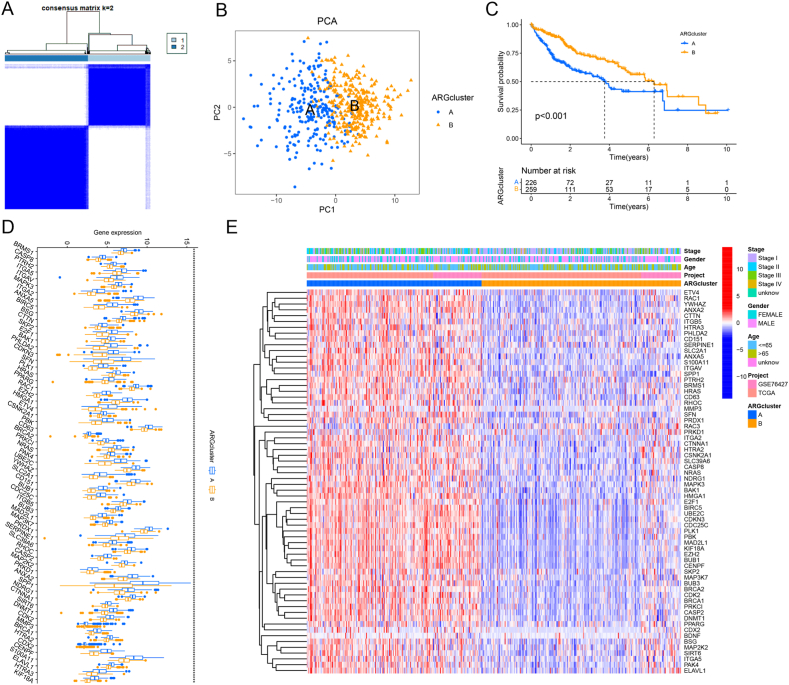
Identification of two molecular subtypes. (A) Consensus matrix heatmap, k = 2. (B) Principal component analysis (PCA) analysis. (C). Kaplan-Meier analysis of overall survival in two molecular subtypes. (D) Relative expression of genes associated with anoikis. (E) Heatmap of clinicopathological features of the two subtypes (cluster A and B).

Dysregulation of ARGs is of significance for disease development and progression [27]. Therefore, ARGs-based identification of molecular subtypes of LIHC can give a better comprehension of the molecular mechanisms underpinning disease progression, thereby aiding in developing novel therapeutic targets. Consequently, we evaluated the differential expressed ARGs in HCC tissues to examine the potential correlation with the prognostic signature. Through the Univariate Cox regression analysis, 66 prognosis-associated ARGs were identified (P < 0.05) (Supplement Fig. 1A). We analyzed the chromosomal positioning of ARGs and the prevalence of copy number variations (CNVs) (Supplement Figs. 1B–C). Combining these data, two distinct LIHC subtypes were determined based on their molecular characteristics (Fig. 1A–B). After prognostic analysis, we found that subtype A was associated with substantial survival disadvantage (Fig. 1C). To gain additional insight, we analyzed the expressions of prognosis-related ARGs in different subtypes of LIHC (Fig. 1D). Notably, the heatmap indicated significant overexpression of most genes in subtype A, as evidenced by the distribution of clinical characteristics (Fig. 1E). We hypothesized that subtype A would benefit more from immunotherapy. We executed both GSEA and GSVA to explore the biological behaviors of these subtypes. The results demonstrated that subtype A possessed more cell cycle pathways than subtype B (Supplement Figs. 2A–D). Based on molecular characteristics, the study classified LIHC into two subtypes and identified a significant survival disadvantage in subtype A, which displayed overexpression of most prognosis-related ARGs.

### 3.2 Identification of anoikis-relevant prognostic risk model

**Fig. 2:**
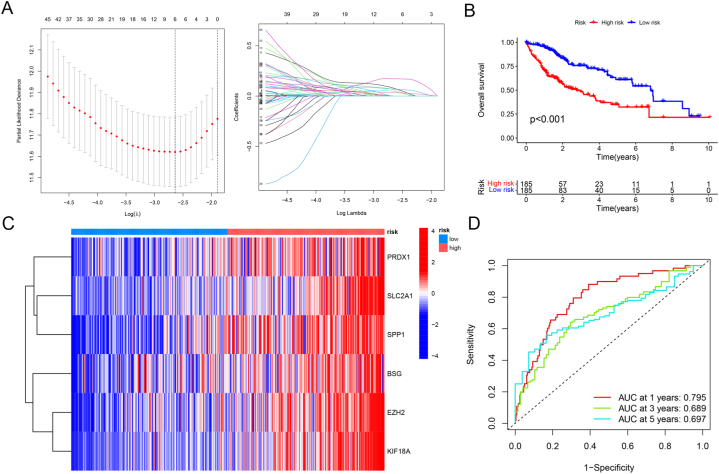
Construction of risk score model. (A) Determination of the number of regulators using LASSO analysis. (B) Survival analysis of LIHC in high- or low-risk groups. (C) Heatmap showing six anoikis prognostic-related genes in different risk groups. (D) ROC curves for the 1-, 3-, 5-year survival prediction.

We developed a risk prediction model for LIHC patients using ARGs. According to survival analysis, low-risk patients had substantially better prognoses than high-risk patients (Fig. 2A–B). Meanwhile, elevated expressions of risk ARGs were associated with an augmented risk of mortality in LIHC populations, as revealed by heatmaps (Fig. 2C) (Table s2). ROC curves in Fig. 2D exhibited the favorable prediction performance of our prognostic risk model. Uni- and multi-variable analyses of independent prognosis demonstrated that the risk score could predict the prognosis independent of gender and differentiation grade, functioning as an independent prognostic indicator (Supplement Fig. 3A). A nomogram was additionally generated using the risk score as a quantitative tool for clinical prognostic prediction (Supplement Fig. 3B). The study designed a reliable ARGs-based prognostic prediction model to assess the risk of LIHC patients; this model may function as an independent prognostic indicator and a quantitative tool to determine patient prognosis in clinical practice.

### 3.3 Immune cell infiltration in LIHC patients at different risks

**Fig. 3:**
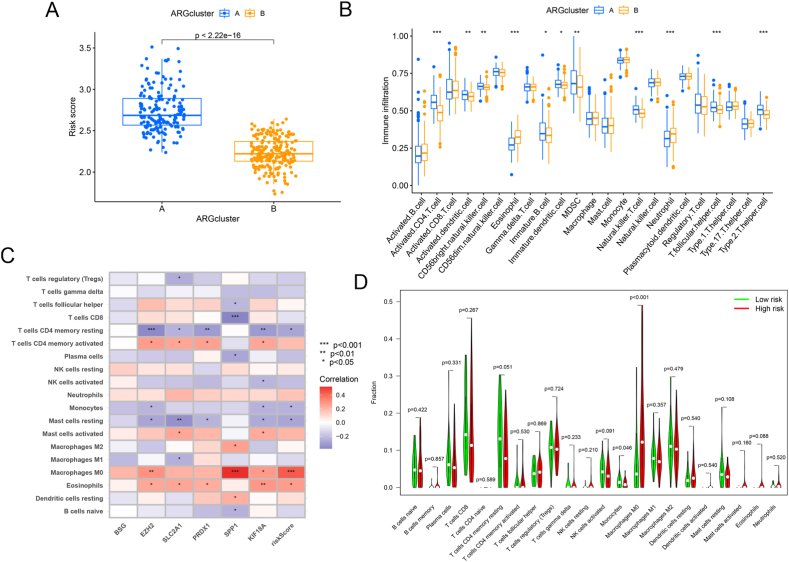
Immune cell infiltration analysis. (A) Differences in risk scores between the two molecular subtypes. (B) Landscape of immune cell infiltration in the two subtypes. (C) Heatmaps of correlation analysis of risk scores and immune cells. (D) Difference analysis of immune cell infiltration in different risk groups.

We verified the relation between the risk score and molecular subtype and revealed significant subtype-specific differences (Fig. 3A). Additionally, ssGSEA demonstrated substantial differences concerning immune cell infiltration between distinct subtypes, such as activated CD4 T cells, activated dendritic cells, CD56 bright natural killer cells, eosinophils, immature B cells, immature dendritic cells, myeloid-derived suppressor cells (MDSCs), natural killer T cells, neutrophils, T follicular helper cells, and Type 2 T helper cells (Fig. 3B). Moreover, CIBERSORT analysis revealed that eosinophils and macrophages M0 shared a positive association with the risk score, while T cells CD4 memory resting, monocytes, and mast cells resting were inversely linked to the risk score (Fig. 3C–D), indicating a possible association between the risk score and immune-related characteristics. Further, the expressions of these three hub genes in LIHC were elucidated using the single-cell transcriptomic dataset (Supplement Figs. 4A–H). The study reported a significant correlation of the risk score with molecular subtype as well as the distinctions in immune cell infiltration, using ssGSEA analysis. Furthermore, CIBERSORT analysis indicated a possible association between the risk score and immune-associated characteristics.

### 3.4 The relationship between CAFs and LIHC prognosis

**Fig. 4:**
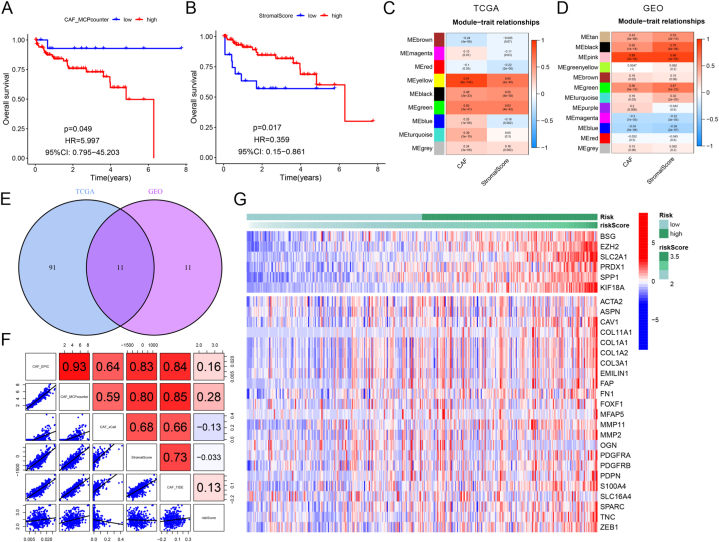
The role of anoikis-related genes (ARGs) in cancer-associated fibroblasts (CAFs). (A) Survival analysis according to CAF score in MCPcounter software. (B) Survival analysis according to StromalScore in MCPcounter software. (C–D) WGCNA analysis of module eigengenes. (E) Venn diagrams showing the intersection genes between the TCGA-MEyellow and GEO-MEpink module eigengenes. (F) The correlation between risk scores and CAF-related genes. (G) Heatmaps of CAF-related genes and ARGs.

CAFs are identified as the prevalent components in the stroma of LIHC [28]. Consequently, we investigated the mechanisms underlying the influence of CAF scores on the prognosis of LIHC patients. First, the CAF score in LIHC was evaluated utilizing the R packages "xCell," "TIDE," "MCPcounter," and "EPIC". Patients with low CAF scores had a more favorable prognosis than those with high CAF scores in the survival analysis (Fig. 4A–B, Supplement Fig. 5A). Next, we determined the relationship between clinical parameters and CAF scores. We conducted WGCNA on TCGA and GEO cohorts to identify the key modules most strongly correlated with clinical traits. The results unveiled that TCGA-MEyellow and GEO-MEpink modules were most substantially associated with CAF score (Fig. 4C–D, Supplement Figs. 5B–C). Fig. 4E depicts a Venn diagram illustrating the intersection of the module genes (Table s3). We subsequently testified the correlation between risk ARGs and CAFs in LIHC. As illustrated in Fig. 4F–G and Supplemental Fig. 5D, risk ARGs were positively relevant to CAFs and CAF-related genes. We also examined the relationship between TMB and CAF score and found no relation between CAF score and mutational load (Supplement Figs. 6A–D). The study explored the impact of CAF scores on LIHC prognosis and identified key modules substantially associated with CAFs.

### 3.5 Cell-to-cell signaling and interaction revealed by single-cell RNA-seq

**Fig. 5:**
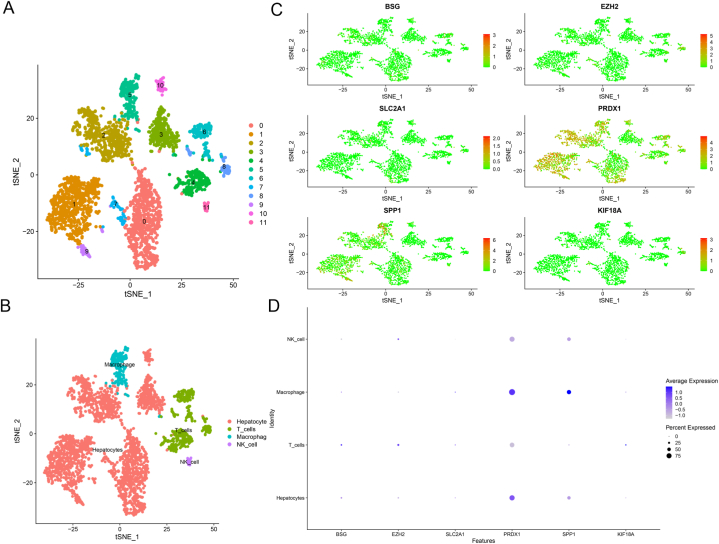
Single-cell RNA sequencing reveals expressions of ARGs in single cells. (A–B) tSNE clustering of single-cell expression profiles based on GSE146115. (C–D) Expressions of ARGs in single cells.

**Fig. 6:**
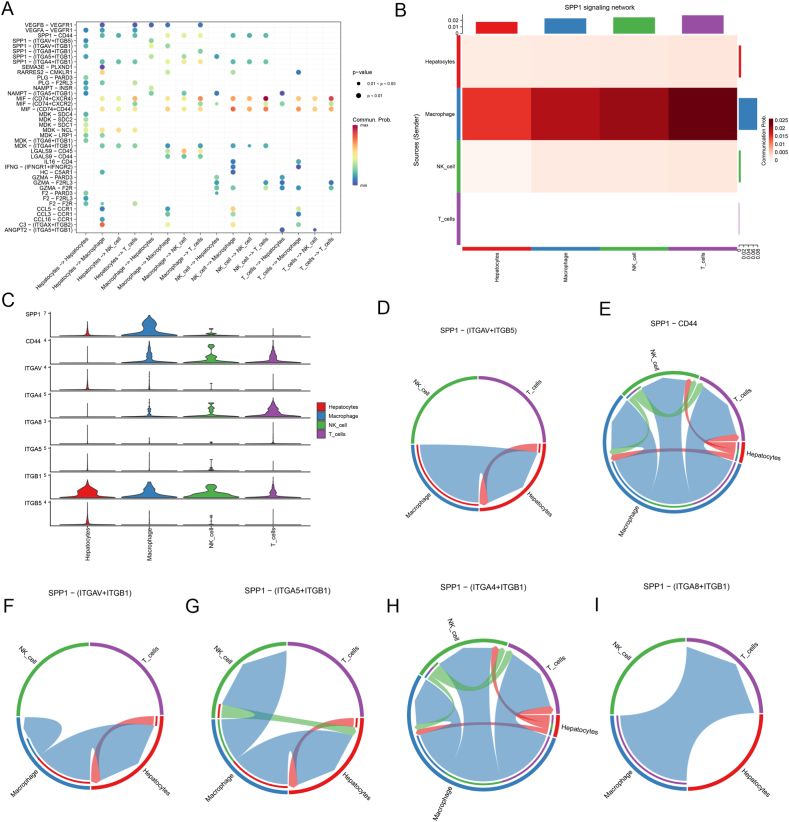
Integrated analysis reveals the pivotal interactions between immune cells. (A) Bubble diagram of cell communication signaling pathway. (B) The role of the SPP1 signaling pathway in cell-cell communication. (C) The expression of SPP1 signaling pathway hub genes in different immune cells. (D–I) SPP1-mediated receptor-ligand pair interactions between immune cells.

After examining individual cells, it was found that the expressions of ARGs vary significantly in immune cells, indicating the critical implication of risk ARGs in microenvironmental mechanisms of tumor progression (Fig. 5A–D). A CellChat analysis on a dataset from the GEO database (GSE146115) was utilized to investigate the possible interactions between distinct immune cells. CellChat is a repository of validated molecular interactions comprising 2021 receptor-ligand interactions. Numerous biological processes, encompassing secreted signaling, ECM-receptor, cell-cell contact, and heterodimers, are dependent on cell interactions. Most ligands and receptors were identified through literature search (Supplement Fig. 7A). CellChat identifies the primary characteristics of intercellular communication in a given scRNA-seq dataset and predicts the pathways that are presently understudied. Our findings demonstrated that macrophages, NK cells, T cells, and hepatocytes interact closely, with receptor-ligand interactions playing a role (Fig. 6A, Supplement Figs. 7B–C) (Table s4). Nonetheless, the intensity of cell-to-cell communication differed among various immune cells (Supplement Figs. 7D–G). The contribution of each ligand-receptor pair to the overall signaling pathway was subsequently visualized. Our investigation revealed that SPP1 was essential for cellular interactions (Fig. 6B) (Table s5). The SPP1 pathway involved sender, receiver, mediator, and influence (Supplement Fig. 7H). Furthermore, the expressions of receptor-ligand pairs (CD44, ITGA4, ITGB1, SPP1, ITGAV, ITGB4, ITGB5, and ITGB8) in immune cells were determined (Fig. 6C). Among them, the ligand-receptor pairs played an important role in the interaction between immune cells within the tumor microenvironment (Fig. 6D–I, Supplement Fig. 7I). Individual cells were examined to evaluate the significance of risk-associated genes in the microenvironmental mechanisms of tumor progression. CellChat analysis revealed potential interactions between immune cells and demonstrated the crucial role of SPP1 in cellular interactions.

### 3.6 Immunotherapeutic responses and chemotherapeutic sensitivity predicted risk subgroups

**Fig. 7:**
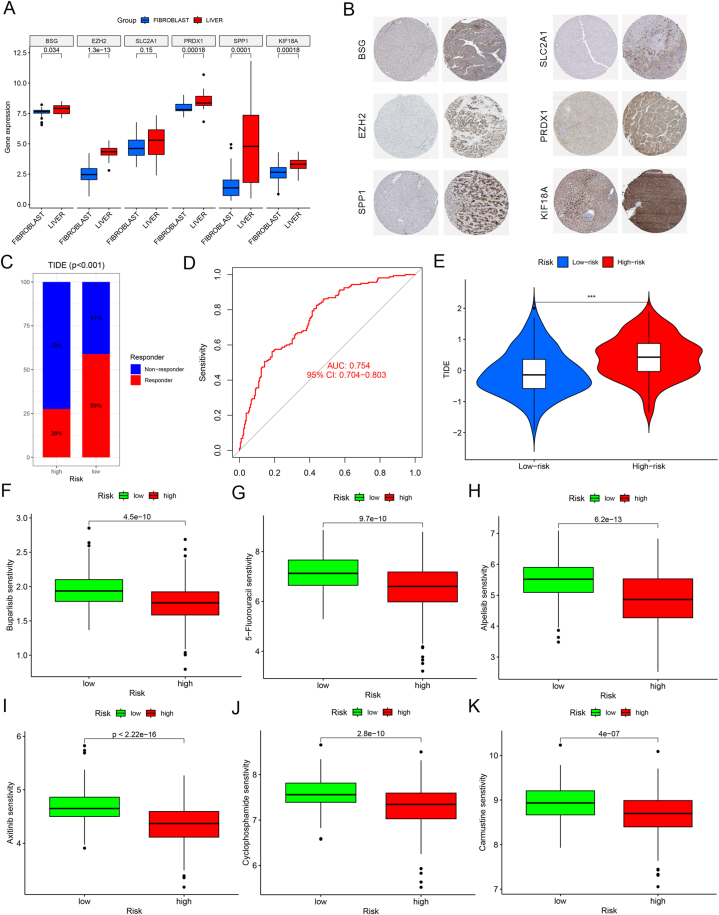
Expression profiles of ARGs and drug sensitivity analysis. (A) Expression profiles of ARGs in CCLE database. (B) Expression profiles of ARGs in HPA database. (C–D) TIDE valuates the immunotherapy response. (E) Immunotherapy response differences between high- and low-risk groups. (F–K) Drug sensitivity analysis in different risk groups.

To validate the expressions of the six risk genes at the translational level, we analyzed their expressions in the Cancer Cell Line Encyclopedia (CCLE) (https://portals.broadinstitute.org/ccle) and Human Protein Atlas (HPA) databases, which revealed highly significant expressions of all genes in LIHC (Fig. 7A–B). Subsequently, we examined the response of LIHC patients towards immunotherapy and chemotherapy drugs. Using the TIDE algorithm, the high-risk population was found to be immunotherapy-tolerant, whereas the low-risk population was sensitive (Fig. 7C–E). Using the "oncoPredict" R package, we anticipated the chemotherapeutic sensitivity for the high-risk and low-risk populations to determine potential chemotherapy drugs. Accordingly, high-risk LIHC sufferers were more sensitive to Buparlisib, 5-fluorouracil, Alpelisib, Axitinib, Cyclophosphamide, and Carmustine (Fig. 7F–K).

## 4 Discussion

Anoikis refers to a unique form of apoptotic cell death occurring consequentially to deficient cell-matrix interactions [27], which is recognized as an important mechanism for maintaining tissue structure and preventing cancer metastasis. ARGs participate in mediating the interaction between cells and extracellular matrix. Accumulating studies have revealed that abnormally expressed ARGs contribute to the onset, progression, and metastatic processes of tumors [29,30]. For example, in breast cancer, dysregulation of ARGs has shown associations with tumor aggressiveness and poor prognosis [2,[31], [32], [33]]. Similarly, in colorectal cancer, ARGs have been linked to tumor invasion, metastasis, and chemoresistance [[34], [35], [36], [37]]. Moreover, ARGs have been identified as potential biomarkers for forecasting clinical outcomes and treatment responses of lung cancer patients [[38], [39], [40], [41]].

The results of our study shed light on several key aspects of LIHC by utilizing various bioinformatics tools and analyses. Firstly, we successfully identified distinct molecular subtypes of LIHC based on ARGs, providing insights into the disease development and progression. Through comprehensive analysis, we discovered that subtype A displayed significant overexpression of prognosis-related ARGs and was associated with a substantial survival disadvantage, indicating the potential of these ARGs as targets for therapeutic intervention. Moreover, the development of an anoikis-related prognostic risk model based on ARGs allowed for accurate prediction of patient prognosis, where the risk score was an independent prognostic parameter. This model holds promise as a quantitative tool for clinical practice, facilitating better risk assessment and patient management. Furthermore, our investigation into immune cell infiltration in LIHC patients at different risks revealed significant subtype-specific differences and immune cell compositions. Notably, we observed associations between risk scores, molecular subtypes, and immune-related characteristics, providing potential implications for immunotherapy strategies. The findings generated in this research are consistent with previous literature demonstrating the significance of ARGs in cancer development and progression [5,41].

Furthermore, our identification of two ARGs-based molecular subtypes of LIHC is consistent with previous molecular subtypes of LIHC identified on the strength of a variety of molecular markers [6]. Our study revealed variations in immune cell infiltration between subtypes, indicating a potential connection between risk score and immune-related characteristics. Moreover, single-cell RNA sequencing revealed intricate cell-to-cell signaling and interactions within the tumor microenvironment of LIHC. Our findings highlighted the importance of risk-associated genes in mediating these interactions, particularly through pathways involving SPP1. The comprehensive exploration of cellular communication within the tumor microenvironment enhances our comprehension of LIHC pathogenesis and potential therapeutic targets.

Additionally, our analysis of CAFs and their impact on LIHC prognosis identified key modules strongly associated with CAFs, providing insights into the role of tumor microenvironment in disease progression. CAFs exhibit high heterogeneity, which is manifested explicitly in the substantial subpopulation of CAFs, as well as the juxtaposition of pro-carcinogenic and anti-carcinogenic roles [9,42]. In breast cancer, CAFs expedite tumor growth and metastasis by secreting growth factors and extracellular matrix proteins [43]. In addition, CAFs can regulate signaling pathways such as TGF-β and CXCL12/CXCR4 to enhance the resistance to chemotherapeutic drugs and targeted drugs. CAFs inhibit immune responses, thereby promoting tumor development. By changing the tumor microenvironment, such as promoting angiogenesis and altering matrix stiffness, CAFs provide a suitable environment for tumor growth [44,45]. In pancreatic ductal adenocarcinoma, CAFs increase the resistance to chemotherapeutic drugs by secreting factors such as IL-6 [46]. CAFs also secrete inhibitory factors such as IL-8 to inhibit tumor growth and metastasis [47,48]. In non-small cell lung cancer (NSCLC), CAFs enhance the sensitivity to targeted drugs like EGFR-TKI by secreting insulin-like growth factor (IGF) and IGF-binding proteins (IGFBPs). Meanwhile, CAFs create a favorable growth environment for NSCLC by influencing the chemical, mechanical, and immune properties of the tumor microenvironment, [49]. Moreover, we identified two modules, TCGA-MEyellow and GEO-MEpink, most substantially associated with CAFs and indicated more satisfactory prognoses in patients with low CAF scores. Previous studies demonstrating the significance of CAFs in cancer development and progression support the utilization of CAF scores to predict LIHC prognosis in the current research [50,51]. The innovation of this study is the ARGs-based identification of molecular subtypes of LIHC, which provides a great understanding of the molecular mechanisms underlying the disease progression and confers novel therapeutic targets. This study also demonstrates the potential of ARGs and CAF scores as independent prognostic indicators for LIHC patients. Lastly, our study evaluated the immunotherapeutic responses and chemotherapeutic sensitivity in different risk subgroups of LIHC patients. High-risk patients were found to be immunotherapy tolerant, while low-risk patients exhibited sensitivity to certain chemotherapy drugs, suggesting the importance of personalized treatment based on molecular risk profiles.

While our study utilizes extensive bioinformatics tools and analyses to explore LIHC, experimental validation can offer valuable confirmation of the findings. Future studies should consider incorporating experimental validation, such as using laboratory experiments to confirm the expression patterns of identified genes or predicted interactions between immune cells in LIHC. Additionally, functional experiments, such as in vitro or in vivo studies, can validate the roles of identified genes or pathways in LIHC progression, metastasis, or therapeutic responses. Integrating experimental validation is warranted to strengthen the robustness and biological relevance of our findings, further enhancing the understanding of LIHC biology and potential therapeutic targets. To sum up, our research gives valuable insights into the molecular landscape, immune microenvironment, and therapeutic responses in LIHC.

## 5 Conclusion

In summary, this study successfully classified liver cancer patients into different molecular subtypes and risk groups by utilizing biological biomarkers such as ARGs and CAFs and revealed significant disparities in immune cell infiltration, cell cycle, and other aspects. The findings drawn from this study provide new ideas and strategies for the treatment of liver cancer.

## Ethics approval and consent to participate

Not available.

## Funding statement

None.

## Data availability statement

The liver hepatocellular carcinoma (LIHC) gene expression data and clinical data GSE76427 were from GEO database (http://www.ncbi.nlm.nih.gov/geo/), and all TCGA-LIHC data, including mutation, CNV, mRNA expression, and clinical data, were downloaded from the TCGA database (https://tcga-data.nci.nih.gov/tcga/). The present single-cell analysis dataset was obtained from the GSE146115. Additionally, the TISCH database was used to analyze the correlations between the expressions of three hub genes and the infiltration of immune cells (http://tisch.comp-genomics.org/). The HPA database was used to determine the protein expressions of six risk genes via immunohistochemistry (IHC) staining and the IHC images were obtained from the HPA database (https://www.proteinatlas.org/).

## CRediT authorship contribution statement

**Meng Sun:** Writing – original draft, Visualization, Validation, Supervision, Conceptualization. **Jiangtao Bai:** Resources, Project administration, Methodology, Investigation. **Haisong Wang:** Validation, Project administration, Methodology, Investigation. **Mei Li:** Visualization, Validation, Supervision, Formal analysis, Conceptualization. **Long Zhou:** Methodology, Investigation, Formal analysis, Data curation. **Shanfeng Li:** Visualization, Validation, Supervision, Formal analysis, Data curation, Conceptualization.

## Declaration of competing interest

The authors declare that they have no known competing financial interests or personal relationships that could have appeared to influence the work reported in this paper.
