# Supplementary material for: Unraveling the relationship between anoikis-related genes and cancer-associated fibroblasts in liver hepatocellular carcinoma
Source: Heliyon. 2024 Jul 29;10(15):e35306. doi: 10.1016/j.heliyon.2024.e35306 (PMC11334810; doi:10.1016/j.heliyon.2024.e35306)
Supplement: Multimedia component 6 [file mmc6.docx]

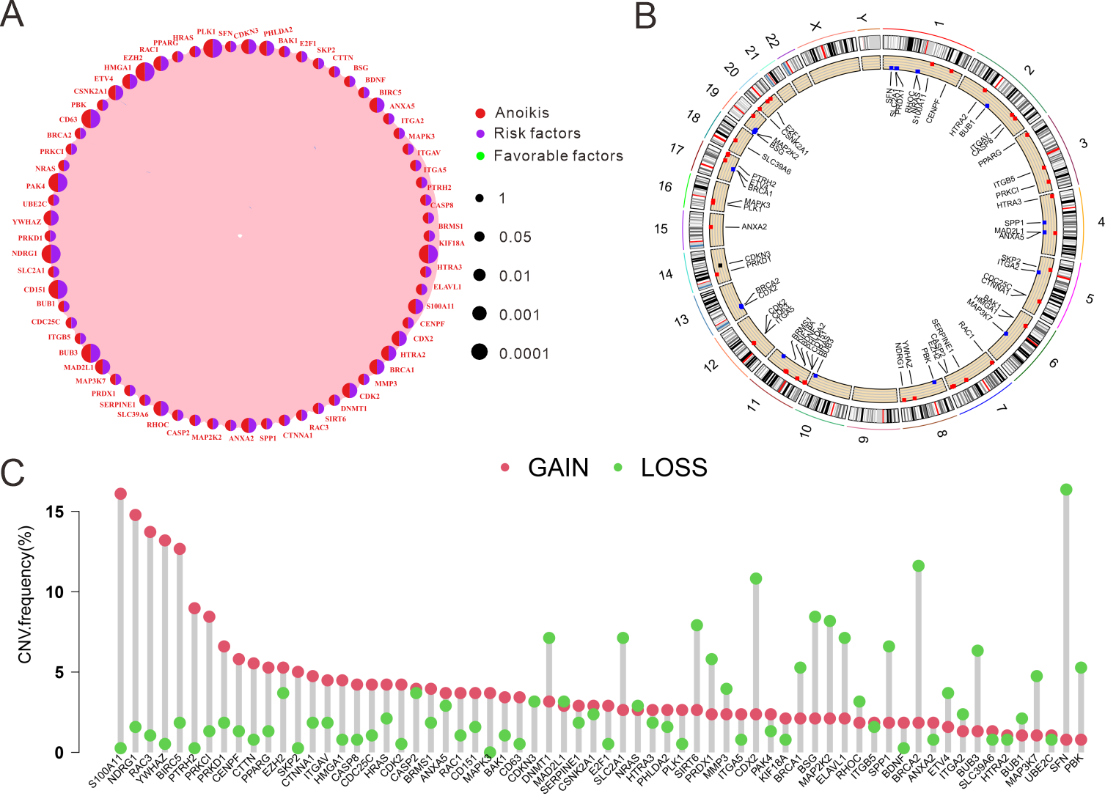


Supplement Figure 1. Analysis of ARGs. (A) The network of ARGs described the combined interactions and prognostic significance. (B) The location of ARGs on the human chromosome. (C) The CNV frequency analysis of ARGs.


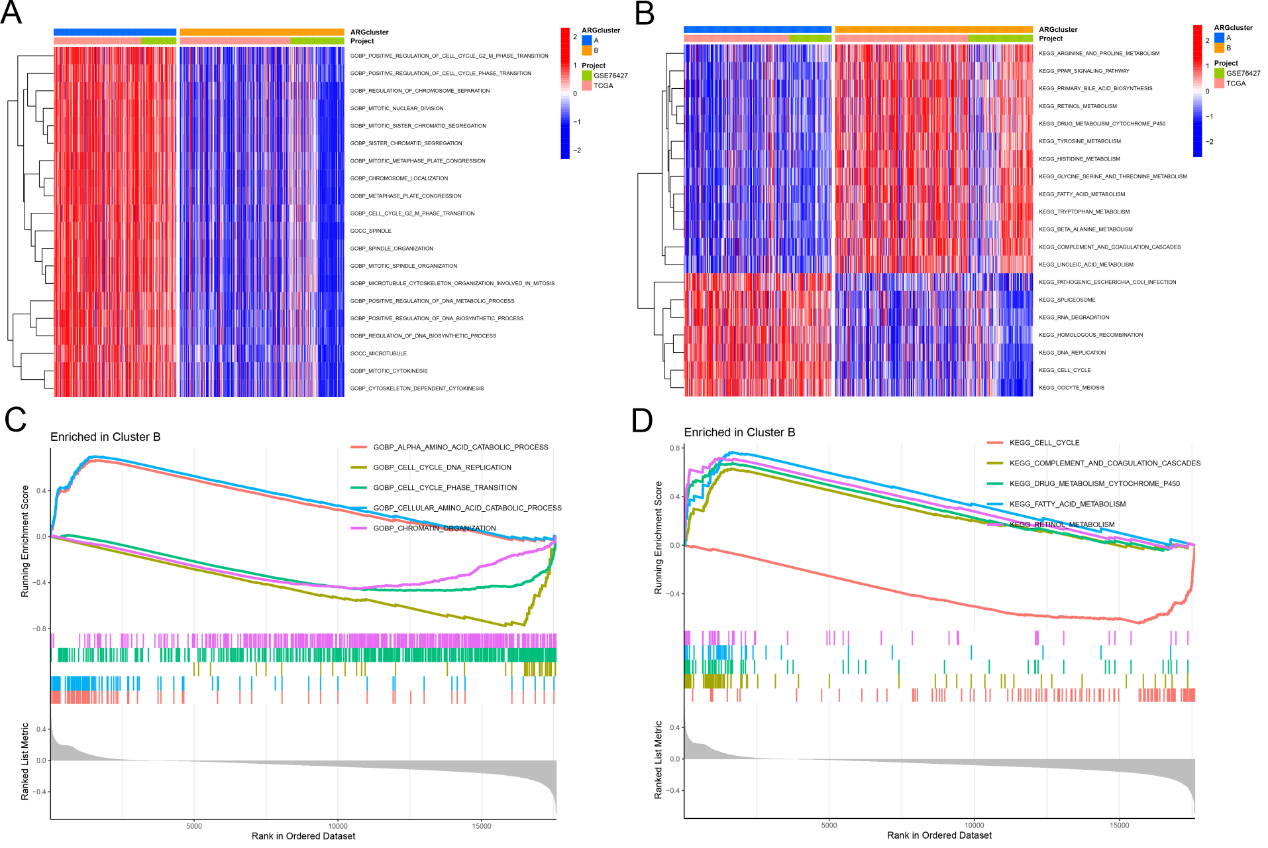


Supplement Figure 2. KEGG and GO pathway analysis. (A) GSVA in two molecular subtypes using GO genesets. (B) GSVA in two molecular subtypes using KEGG genesets. (C) GSEA analysis in cluster A subtype. (D) GSEA analysis in cluster B subtype.


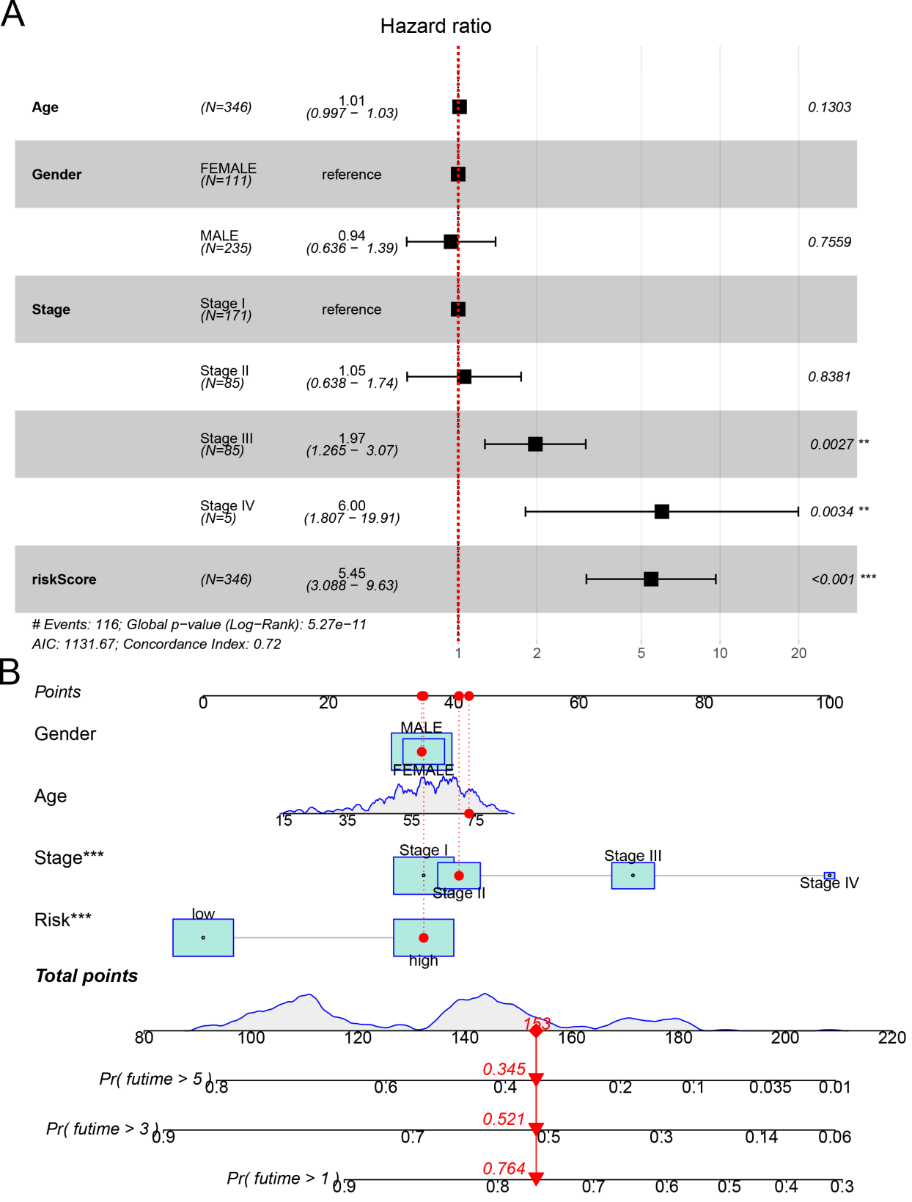


Supplement Figure 3. The predictive performance of risk score and other clinicopathological parameters. (A) Multivariate Cox regression analysis of risk score and clinical features related to prognosis. (B) Prognostic nomogram analysis for LIHC.


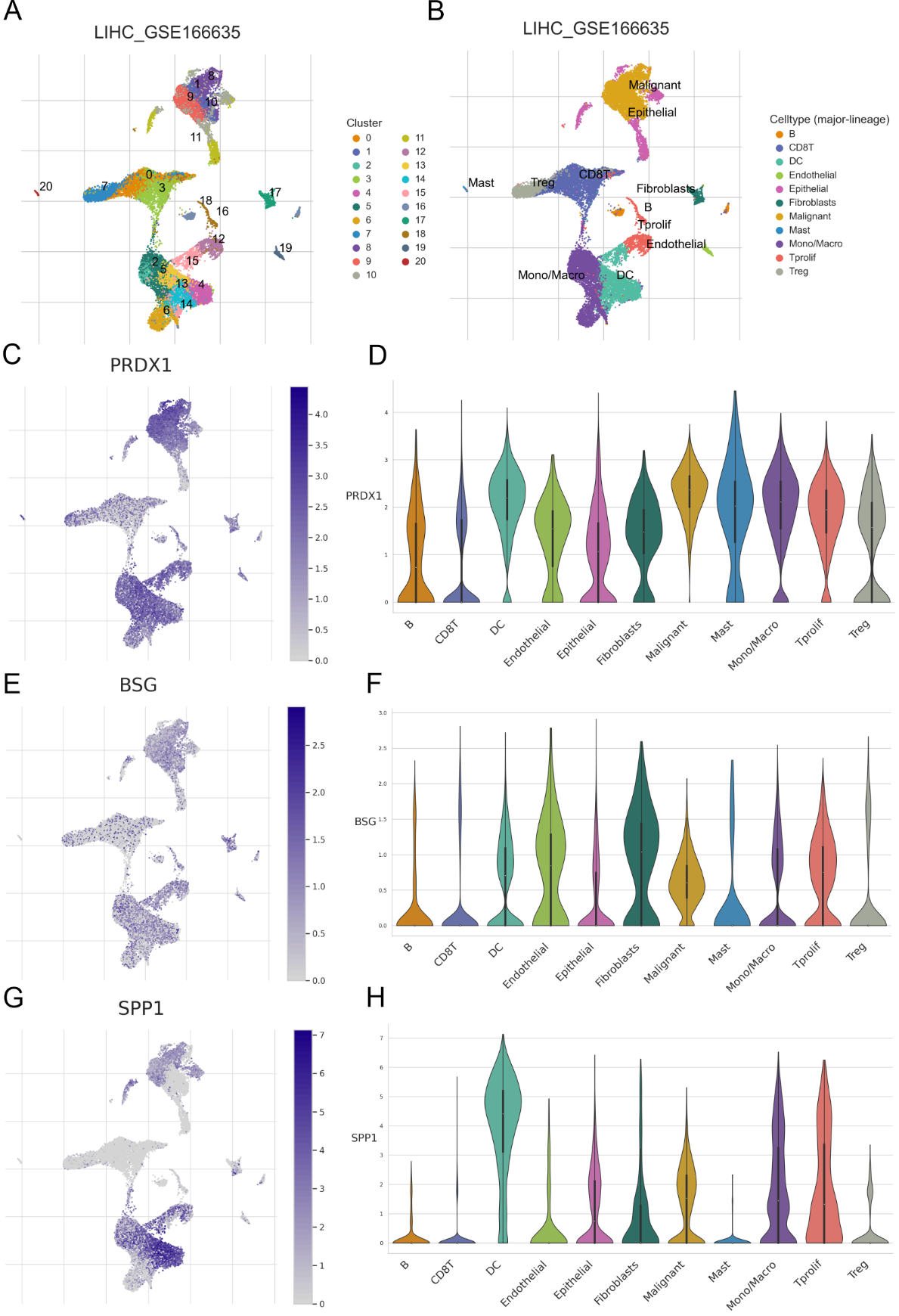


Supplement Figure 4. Single-cell RNA sequencing reveals expressions of ARGs in single cells. (A-B) tSNE clustering of single-cell based on GSE166635. (C-H) Expressions of ARGs in single cells.


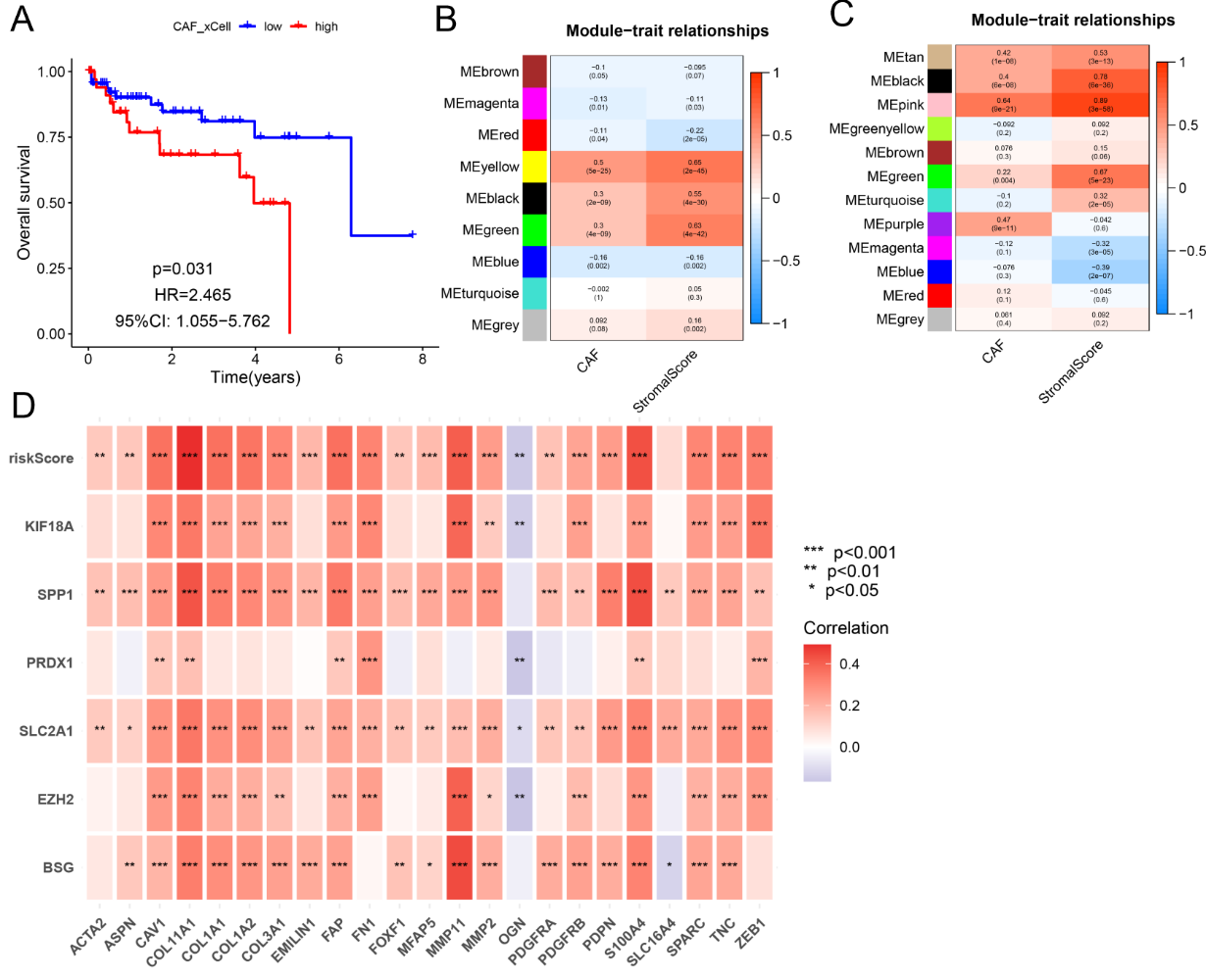


Supplement Figure 5. Analysis of CAF scores based on xCell software. (A) Survival analysis according to CAF scores in xCell software. (B-C) WGCNA analysis of module eigengenes. (D) The correlation between risk score, ARGs, and CAF-related genes.


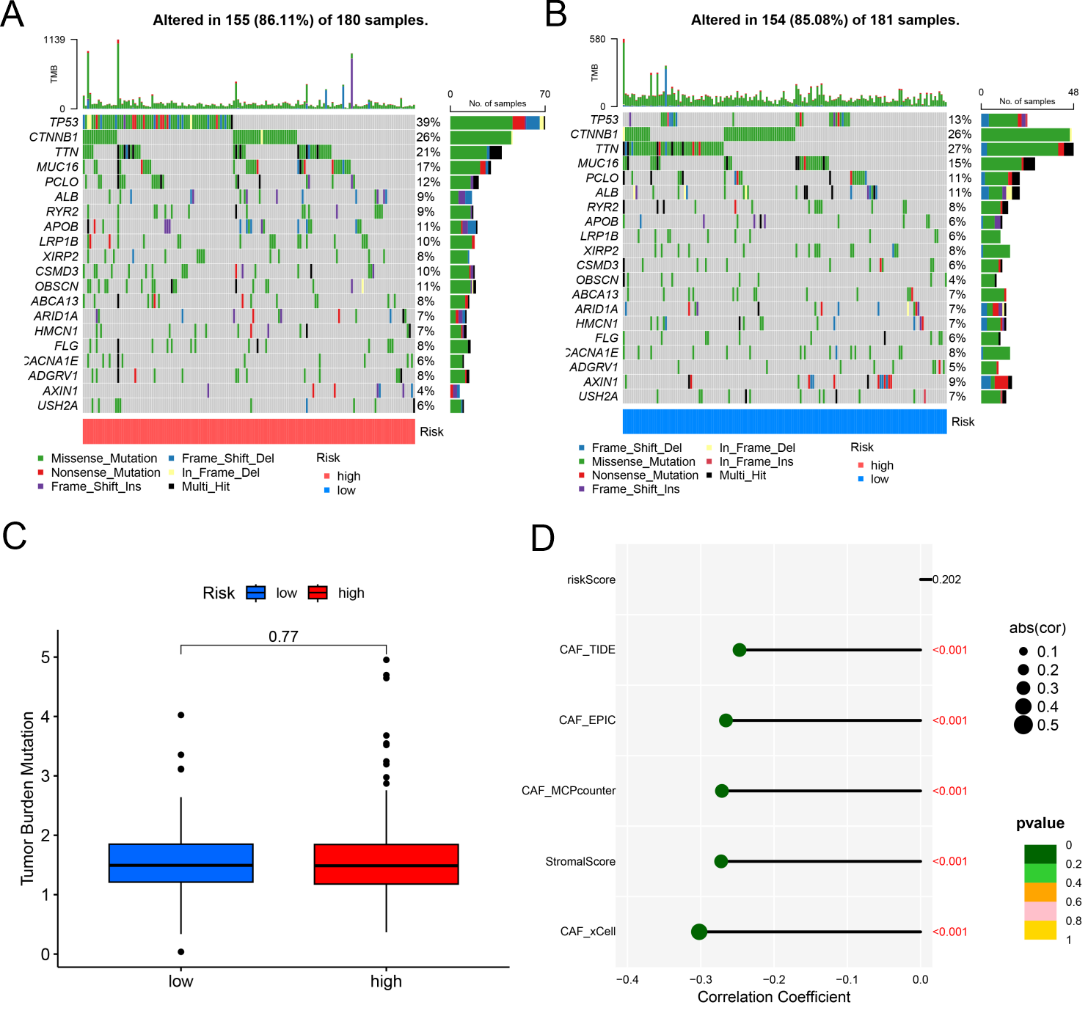


Supplement Figure 6. Mutational analysis of ARGs in high- and low-risk groups. (A-B) Analysis of gene mutation in high- and low-risk groups. (C) Analysis of tumor mutational burden (TMB) in high- and low-risk groups. (D) Spearman correlation analysis between risk score and CAF score.


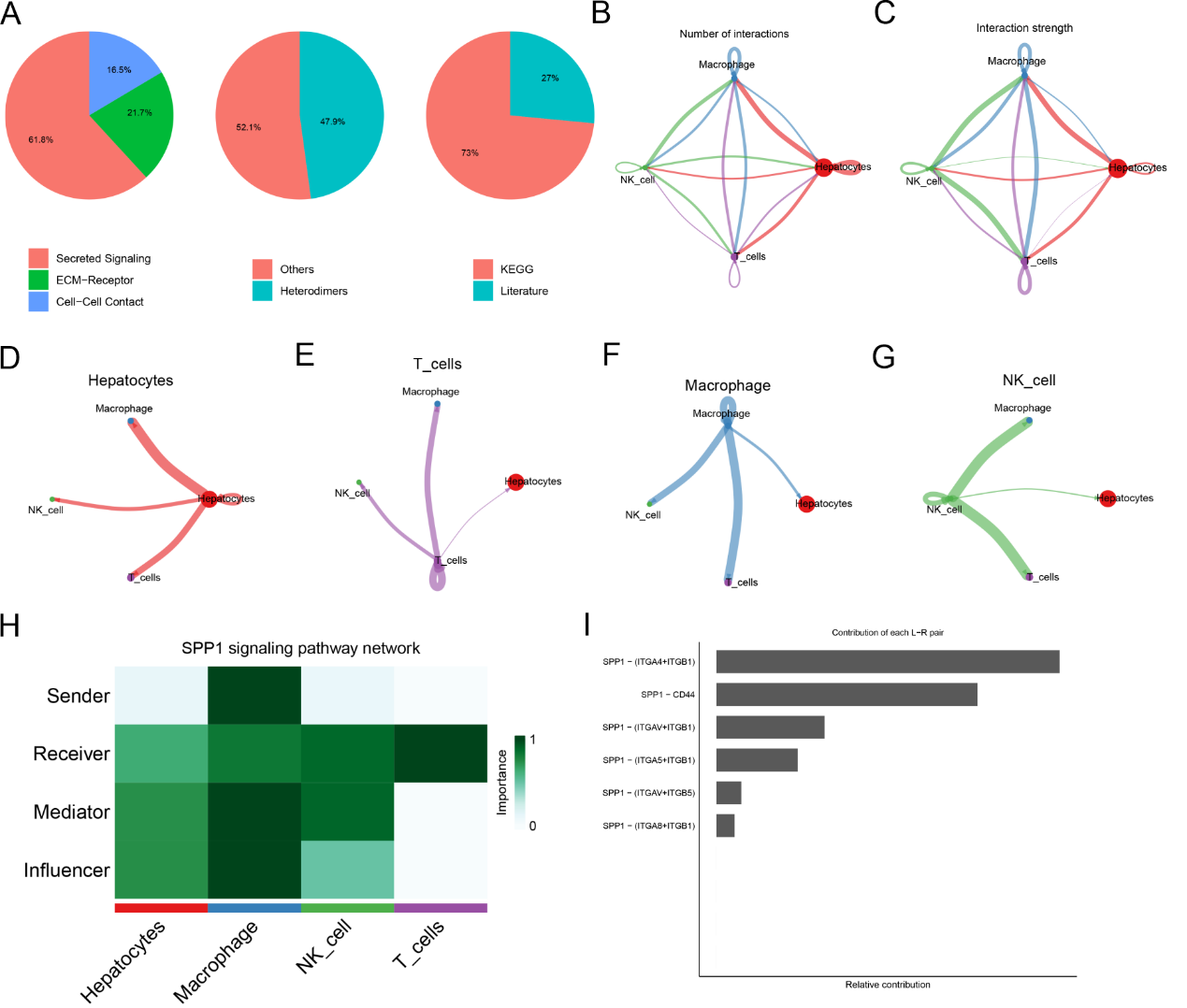


Supplement Figure 7. Integrated analysis reveals the pivotal interactions between immune cells. (A) The cells interact with receptors and ligands. (B) The number of cell interactions. (C) The strength of cell interactions. (D-G) Individual cells interact with each other. (H) Analysis of the interaction mode of SPP1 signaling pathway in cell communication. (I) SPP1-mediated receptor-ligand pair interactions between immune cells.

Table s1. ARGs were obtained from GeneCards and Harmonizome.

Table s2. The expressions of risk ARGs among high and low LIHC risk groups.

Table s3. A Venn diagram illustrating the intersection of the module genes.

Table s4. The primary characteristics of cell-to-cell communication in LIHC.

Table s5. SPP1 signaling pathway in cellular interactions.
